# A Case for Moving Beyond Verbal Labeling in Facial Expression Perception Research

**DOI:** 10.1007/s42761-025-00341-w

**Published:** 2026-02-02

**Authors:** Reginald B. Adams, Max Weisbuch

**Affiliations:** 1 Department of Psychology, The Pennsylvania State University, University Park, PA USA; 2 Psychology Department, University of Denver, Denver, CO USA

**Keywords:** Facial expressions, Emotion labeling, Functional theory, Vision perception

## Abstract

Dating back to Darwin’s original treatise on emotions in humans and in animals (Darwin, 1872/1997), theories of emotion—from classic to contemporary—are built on the premise that emotions, and our expression *and* perception of them, serve functional purposes. Testing these theories, however, has relied almost exclusively on verbal labeling tasks (again dating back over 150 years to Darwin and extending into the present). Of particular relevance to the current review is that functional theories of emotion expression perception contend that expressions inform specific types of perceiver behaviors and outcomes. These perceiver behaviors and outcomes are central to any functional theory. Yet these same theories rarely measure adaptive behaviors or other outcomes in the perceivers. For reasons expounded upon in this paper, we propose that this approach limits the conclusions that can be drawn. Even in paradigms not employing verbal labeling, facial expression stimuli are generated through verbal labeling methodology. This results in a set of expressions necessarily reflecting lay conceptualizations of emotion expressions, which then drives the science of facial expression perception. This approach is limiting as lay conceptualizations are subject to change over time and thus make it prone to missing the immutable processes and mechanisms underlying expression perception that are central to our science. Finally, we offer some initial recommendations for how we, as researchers in the domain of facial expression perception, can begin to move beyond the use of verbal labeling in our stimulus generation and research paradigms.

Charles Darwin (1872/1997) famously argued that facial expressions of emotion evolved because they once were (if not still are) adaptive to our survival. This *functional approach* to facial expressions directly contradicted then-popular theories of emotion. For instance, Sir Charles Bell, who also studied facial expressions in the 1800s, contended that God endowed us with the capacity of facial expression to convey our emotions to others. The idea that emotion expression evolved through functional adaptions was a radical departure from the assumptions of the day. Fortunately, subsequent theories of emotion and emotion expression embraced Darwin’s functional account, including William James’s embodiment theory of emotion (1884; see also Niedenthal, 2007), Magda Arnold’s cognitive appraisal theory of emotion (1960; see also Scherer, 1984), and Silvan Tomkins’s noncognitive affect theory (i.e., discrete emotions, 1962; 1963: see also Ekman, 1973; Keltner & Oatley, 2022). Although varying on some basic assumptions, each of these approaches nonetheless agreed that emotions and their concomitant expressions are functionally adaptive.

Where Darwin argued for the evolutionary function of facial expression, his theory made no evolutionary claims about functions of perceiving emotion expressions, just that we can perceive them in a culturally universal manner. In this essay, we critically review contemporary approaches to understanding the *functions* of facial expression perception. We aim to clarify the concepts and methods used within this domain with recommendations for producing a more coherent science.

## Defining our Terminology

It is first important to clarify our usage of the terms facial expression and facial expressions of emotion, particularly in the context of the functional emphasis we put on expression production and perception. “Facial expressions” can be defined as facial configurations that reliably respond to and influence the environment. This definition is not meant to eschew the psychological processes that give rise to these configurations. Instead, this definition illustrates that the term “expression” is simply conventional and actually implies a theoretical position, i.e., that the configuration of a face is caused by emotion (which scientists seem to understand as a constellation of physiology, cognition, and behavior); however, we suggest there are at least two reasons that functional approaches to perception can be agnostic with respect to “what” is expressed.

First, perceivers only have direct access the physical display of a face—human perceivers do not have extrasensory perception, so they are left with a physical (facial) display. Perceivers’ adaptive responses to these facial displays need not recapitulate the processes that caused the display but rather need to promote the perceiver’s well-being in response to that particular display. It is possible that perceivers achieve the latter function via the former but doing so essentially represents a lay theory, not a definition of facial expression.

Second, evidence suggests there is little if any association between facial emotion and physiology (e.g., Barrett et al., 2019). Critically, functional approaches need not assume there is an association. For instance, in work employing a social functional approach to facial expression *production*, Wood et al. (2017) state that “our social functional account is agnostic about the feeling states underlying an expression, instead seeking to identify common social consequences” (p. 5). Similarly, Adams et al.’s (2017) social functional approach to facial expression *perception* emphasizes perceived behavioral forecasts and action-perceptions, not emotion per se (see also Gibson, 1979; Zebrowitz, 1997). Ultimately, we use the term facial expression to refer to facial displays that generate reliable and functional responses in their environment.

A final note on this definition regards usage of the phrase “facial expressions of emotion”. This is another phrase that we believe has been conventionalized to again imply that a specific mental state is the cause of a facial display. Here, facial expressions of emotion refer to facial configurations that people consensually label with an emotion word. Again, it is possible that those consensual labels always reflect an emotion that causes the facial display, but this is a theoretical position—those words may instead reflect practical labels for facial displays that reliably reflect the expressor’s behavior tendencies. We return to this discussion later, but the key point for this article is that facial expressions are regarded as facial displays that consistently appear under particular environmental conditions.

## Facial Expression Perception

Darwin argued that facial expressions are the product of behaviorally adaptive responses to recurrent survival obstacles but did not evolve to signal social information to others. Current opinion today, however, stresses that perceivers learned to take advantage of expressions’ predictive value to gain a survival advantage, i.e., as a signaling system: if facial expressions evolved to serve an adaptive function, it certainly stands to reason that the ability to perceive and react to them also serves an adaptive function.

Seeing another’s expression can cue us into potential reward (food) or harm (predator) in the environment, and thus ready us for an appropriate behavioral response. As such, from a social functional perspective, when we perceive an emotion expression, we are arguably more tuned to the behavior or event it forecasts than to detecting a specific emotion per se (e.g., Adams et al., 2017). Despite wide disagreement over whether emotional displays are universal or culturally shaped, cognitive or biologically mediated, directly linked to felt emotion or not, contemporary theories do tend to agree that facial expressions act to forecast an organism’s behavioral tendencies (e.g., Ekman, 1973; Izard, 1971; Fridlund, 1994; Frijda & Tcherkassof, 1997; Russell & Fernández-Dols, 1997; Weisbuch & Adams, 2012).

Despite the prevalence of such “functionally-predictive” approaches to facial expression perception, there is a relative dearth of empirical evidence that facial expressions *are* actually predictive of an emoter’s behavior. Does facial anger precede aggressive acts? Does facial fear precede avoidant acts? From Darwin’s perspective, such evidence is irrelevant—we inherited facial responses from our animal ancestors and there is no need for them to be predictive *in the present* (see also, Barrett, 2013 for review). But, for the most part, classic and contemporary theories alike highlight the functional nature of facial expressions and in turn facial expression perception. This assumes that facial expressions both influence the behavior of those around us (e.g., Niedenthal & Brauer, 2012) and meaningfully forecast our own likely behavior to others (e.g., Weisbuch & Adams, 2012). In the animal literature, emotional expression is studied through behavioral observation with this assumption in mind, with no need to make assumptions about corresponding underlying subjective states that might be involved. Few studies, however, have examined emotional expression in humans in this way.

One notable exception to the above contention is Robert Provine’s work on smiling and laughter (2001). Following Jane Goodall’s method of studying chimpanzees, he and three research assistants employed an observational method to study human laughter. After observing over 1,200 encounters of people interacting face-to-face in public settings, they found that spontaneous bouts of smiling and laughter had little to do with felt amusement and more to do with what Provine concluded was a “nonlinguistic role in social bonding solidifying friendships and pulling people into the fold. You can define ‘friends’ and ‘group members’ as those with whom you laugh” (p. 47). We highlight this example to demonstrate the utility of decoupling expressive behaviors from verbal reports of their corresponding subjective states and utilizing paradigms that examine downstream function on behavioral, perceptual, psychological, and physiological outcomes. Decoupling emotion expressions from their presumed emotional states also allows for comparative research and models (e.g., Panksepp, 2015). Further, labeling emotions is also not a default way of understanding or describing facial expressions in many nonwestern cultures (e.g., Wnuk & Wodowski, 2024), thereby limiting cross-cultural comparison as well.

Yet, beginning with Darwin’s initial inquiry and continuing on into most contemporary research on human emotional expression, the prevailing method used to study emotion expressions perception has been through the verbal labeling of emotions. We contend here that like the animal literature, it is not necessary to make inferences about underlying subjective emotional experiences when studying facial expressions, and in fact there is likely much to be gained by not doing so (see also Adolphs & Andler, 2018, reviewed in more detail below).

## Measuring the Behavioral Component of Emotion Expression Perception

Perceptions of emotion expressions have been examined with a variety of behavioral measures. In this section, we briefly review examples of such studies to illustrate both obvious and non-obvious limitations associated with the field’s emphasis on verbal labeling.

### Emotion Labeling in Studies of Emotion Perception

Speeded reaction times in labeling an emotion expression are often employed to test functional theories of emotion perception (e.g., Adams et al., 2006; Marsh et al., 2005): if you can quickly label an emotion expression, arguably you can more rapidly adapt your behavior to another person’s emotional state. For instance, in one study examining approach and avoidant tendencies elicited by anger and fear expressions, a joystick paradigm was employed (Marsh et al., 2005). In this study, participants had to label faces as quickly and accurately as possible while either pushing the joystick (extension/avoidance, i.e., pushing away) or pulling it (flexion/approach, i.e., pulling toward). They found that participants were faster at labeling anger when pushing the joystick, and fear when pulling it. Although this study advanced theory in an important way by including an important behavioral component, the primary task was still verbal emotion labeling.

This tendency to use verbal labeling as the outcome variable is the case for almost all studies of emotion perception. Yet, the theories that motivate such studies rarely describe the linguistic and visual-linguistic processes that give rise to word identification and generation. There is a vast literature in psychology on visual and spoken word identification that includes detailed computational models for how people recognize and generate words (e.g., Rayner, Li, & Pollatsek., 2007; Sedenberg & McClellan, 1989), including how they label visual stimuli (Lupyan, 2015). Moreover, rendering labels in an experiment may be regarded as verbal communication and if so, Gricean norms (Schwarz, 1999) and related communicative processes may account for responses on the dependent variable (DV). Because functional theories of emotion perception typically fail to elaborate the labeling processes involved in the DV, the impact of the independent variable on the DV may regard *functional labeling* rather than *functional behavior*.

This line of reasoning could be resolved by scientific acceptance that language is a necessary limitation to studies of emotion perception, where the concept of “perception” is understood as perception *that can be verbally communicated*. By this approach, functional theories of emotion perception regard the function of communicating something in response to an emotion expression, which may indeed have critical social implications for the perceiver. However, theorists who are focused on how people adapt to their environments will find this approach unsatisfactory, as adaptation is not limited to labeling things in the environment but rather extends to actions (involving body movement), enduring behaviors (e.g., avoidance) etc.

### Studies Absent Emotion Labeling

Beyond adopting a strictly linguistic framework to emotion perception, some functional approaches to emotion perception have been examined in the absence of emotion labels. For instance, Adams et al. (2006) presented participants with anger and fear expressions in the center of a computer screen that either appeared to pop out or fall back into the screen. Participants were simply asked to indicate if the stimulus was approaching or withdrawing as quickly and accurately as possible, no emotion label applied. In two studies, faces were labeled as approaching more quickly when displaying anger versus fear, and vice versa for faces labeled as withdrawing. We review this study because it includes another type of paradigm used in emotion perception: participants responding rapidly (or to briefly presented expressions) with a labeling response that is *not* an *emotion* label. Yet, even in this case, labeling or word generation processes were still likely shaping the DV and perhaps in a manner that accounts for the results.

Some methodologies have been employed that measure behavioral responses with *no* verbal label, emotion or otherwise, invoked. That is, the DV in these studies is observed behavior. For example, Winkielman et al. (2018) examined how subliminal exposure to facial expressions shape perceivers’ consumption behavior (i.e., drinking or eating). But, even this study utilized facial expressions of emotion that were *validated* through verbal labeling. The same is true of other methods that have been employed to avoid verbal labels. For instance, the method of adjustment (MoA) does not require a verbal response from participants. Rather, participants adjust a facial image (scrolling left or right with their mouse) until it corresponds to a facial emotion they saw a moment earlier. Although participant responses are not verbal in the MoA, their responses are nonetheless limited to the dimension previously identified via verbal labels. Thus, even this example can be understood in terms of the psychological processes that *link* perception to verbal emotion labeling.

## Implications of Verbal Emotion Labeling on Standardized Expressions

As noted above, consensual labels for displayed expressions are typically used by scientists to select expressive stimuli for their studies. A quick perusal of any of the many studies on emotion expression perception will reveal widespread usage of face databases that use verbal emotion labeling as their metric. These consensual labels are *the* validity criterion—notably, this means that facial expression databases are based solely on *face validity*. In other words, if most people label an expression as “anger”, for example, then it *is* an expression of anger. But are these expressions actually the one’s that (a) people display when they are angry and (b) that elicit “functional” responses? There is little existing evidence for the *predictive validity* of emotion expressions (i.e., do they forecast behavior?), the *discriminant validity* of emotion expressions (i.e., do they produce different effects than non-emotional expressions?), or the *convergent validity* of emotion expressions (i.e., do they predict emoters’ physiological responses?). Although existing evidence suggests that expressions and physiological responses are not closely related (Barrett et al., 2019), the lack of evidence for predictive validity (behavior) is critical. For example, if the emotion expressions labeled by laypersons as “angry” are not *predictive* of the emoter’s observable actions, perceivers would have little reason to “adapt” to that expression.

Facial emotion databases have been used widely in psychology, such that scientists use these normed stimuli in their studies, use these stimuli as the basis for “morphing” expressions, and use these stimuli to test “emotional intelligence”. Yet, the methodology for validating these databases arguably presents real limits on the field of emotion perception. Specifically, validation procedures typically require study samples to verbally label a facial expression—each image is indexed by the degree to which participants use a particular emotion label for a particular emotion expression. Although other validation approaches exist, these approaches (e.g., FACS coding; Ekman & Friesen, 1978) are themselves validated by consensual verbal labels for the emotion in the facial image.

Ultimately, any studies using facial images validated from consensus in verbal labels are using materials that are subject to the current critiques, though these critiques are especially relevant to studies that include verbal labels as a dependent measure. Functional approaches to the perception of emotion displays, for example, use a variety of study designs but they typically use stimuli based on verbal consensus and/or require participants to verbally label the expression in terms of emotion. Ultimately, this means that limitations of verbal labeling apply to the vast majority of studies examining perception of emotion displays.

## Why Move Beyond Verbal Labels?

This paper, and special edition, was inspired by a set of workshops that came together to discuss contemporary emotion expression and emotion expression perception science and theory. One of the goals of the workshops was to get expert recommendations for future sets of emotion expression stimulus sets. Our recommendations below are a tall order, but we believe that the emotion community has the technological advances and expertise to begin to put together a more comprehensive set of experimental stimuli, widely available to researchers, through future collaborative ventures.

We argue here that, in many cases, facial expression databases currently available are limited in scope. Specifically, process-oriented and function-oriented theories of emotion perception that utilize facial expression databases unnecessarily limit themselves to processes and functions associated with verbally labeling an expression for its emotion or other properties. For example, imagine a scientist who theorizes that racial identity biases the perception of emotion in a face: people should see anger more quickly in outgroup than ingroup faces. This scientist developed morphs derived from stimuli in a facial emotion database. The study involves brief exposures to facial expressions and requires a binary judgment (e.g., fear/anger) from the participant. Psychometric curves are then generated for cross-race and same-race faces, per participant, and results support hypotheses: participants require less exposure time to label “anger” (but not fear, sadness, or disgust) in cross-race than same-race faces. The scientist then argues that this is evidence that race biases the perception of anger in the face. This conclusion may be accurate but may be limited to perception that is conceptualized as that which can be verbally communicated and readily available in the cultural lexicon. That is, facial emotion was methodologically defined as facial expressions that are normatively labeled as specific emotions.

Adolphs and Andler (2018) offer some solutions to this approach in the domain of emotion in general, rather than perceptions of emotion specifically: we would summarize their position as arguing that emotions are like latent variables that produce a number of independent (and interactive) effects on perception, cognition, subjective feelings, judgment, and behavior. In this work, the authors take affective scientists to task for relying on self-reported feelings to measure emotion and note that the functional output of emotion can include but is *not limited to* labeled emotions. Thus, emotions need not be verbally identified by laypersons but rather defined by a constellation of effects that involve multiple systems. By this approach, we scientists may define *new emotions* that produce a syndrome of effects even if the new emotion is not in the lexicon of laypeople, and we can identify emotions in the cultural lexicon that do not conform to a scientifically defined emotion.^1^

Just as a person’s own emotion need not be limited to subjectively labeled feelings available in the cultural lexicon, perceptions of emotion (and emotion expression databases) need not be limited to subjectively labeled feelings. A given facial expression may elicit cognitive, affective, and/or behavioral responses from perceivers, and such effects may occur in the absence of verbal labeling processes. It is these processes that are at the heart of functional theories of emotion perception that ask: what are the “adaptive” outcomes associated with perceiving a facial display?

This question demands a focus on facial displays as stimulus configurations, where those configurations are not *defined* in terms of some internal state (e.g., “fear”) but simply refer to the configuration. And for functional theories of emotion perception, the critical question regards how encountering such a stimulus configuration shapes a perceiver’s adaptive cognition and behavior. Yet we believe this area of inquiry has, in many cases, put the cart before the horse as some fundamental questions remain unanswered, as detailed below. To abide by space limitations, our suggestions below for the future of emotion perception research focus on two key questions. Even if readers only consider these questions (as opposed to fully endorsing our view), we believe we will have “done our job” in moving the field forward.

### Facial Expressions Versus Facial Expressions of Emotion

Do facial expressions of emotion elicit stronger adaptive responses than do facial expressions that are *not* consensually labeled with an emotion word? This is a simple yet critical question for functional theories of emotion perception: if facial configurations that are consensually labeled with an emotion word do not elicit stronger adaptive responses than other facial configurations, functional approaches to emotion perception may be abandoned in favor of broader theories that focus more generally on perceptions of reliable facial expressions. This question is also one that can be clearly answered via scientific inquiry. Scientists can measure adaptive responses (e.g., visual vigilance, sympathetic nervous system activation) in response to different facial expressions, as in prior work. However, only some of those facial expressions would have been consensually labeled with emotion words compared to other expressions which might resemble those expressions but differ qualitatively. For example, a “fear” expression absent raised eyebrows or a “joy” expression with the addition of furrowed brows both qualify as resembling consensually labeled emotions but are *not* typically labeled with that word.

Rachael Jack and colleagues (2012) innovative approach to emotion perception is an excellent candidate for this approach, as facial expressions can be generated from the bottom-up approach (randomly, if desired) to allow for comparisons across many different types of facial expressions. Ultimately, it seems critical for functional theories to establish that consensually labeled facial expressions of emotion are “special” (or not) in terms of the responses they elicit from perceivers, and not simply because people agree about what these facial configurations should be called.

Regardless of the answer to this question, it will enable scientists to better identify their domain of inquiry, including its scope and limitations. Evidence in favor of the view that consensually-labeled facial expressions *are* special would support a continued focus on those facial expressions that can be labeled with a categorical emotion. Evidence in favor of the view that consensually-labeled facial expressions are *not* special (with respect to the responses they receive) would support a broader approach that does not prioritize facial expressions of emotion but instead seeks to identify those expressions that compel reliable responses in perceivers.

### Emotion Recognition Versus Normative Evaluation

What are people *thinking* when they label facial expressions? To the degree that affective scientists wish to retain traditional validation procedures for facial expression sets, this is a critical question. Typical instructions for participants fail to define specific emotions or emotion in general, and simply ask them to select, rate, or state the emotion in a face as part of validating a facial expression set or as a dependent variable. This procedure leaves considerable interpretive territory for what participants are thinking when they label a facial expression. It is possible that participants are truly making mental state inferences. Research in the domain of Theory of Mind underscores the ability of humans to reason about others mental and emotional states (e.g., Baron-Cohen, 1995), a feat that is presumed to be highly evolved (e.g., Premack & Woldruff, 1978), with certain psychopathological disorders (e.g., autism) being marked by, even defined by, deficits in this ability (see Emery, 2000). That said, given that people also report perceiving facial emotions when viewing written English (i.e., Roman letters, Weisbuch et al., 2020) and when viewing neutral faces (e.g., Montepare & Dobish, 2003) with reliable rates of consensus similar to that for facial expressions, it is also possible that ratings of emotion in faces can reflect communicative norms. In lay terms, participants may be thinking, “how would people label this facial display?” and not “what emotion is this person experiencing?”

This question about the nature of “emotion recognition” sits at the heart of our critique. We have argued that the labels people provide for facial expressions does not necessarily coincide with perceivers’ adaptive responses to those facial expressions. Specifically, the processes involved in labeling an emotion may be independent or weakly related to the many adaptive responses that people (presumably) have to facial expressions. If so, the facial expressions that give rise to consensual labels of emotion may not be those that give rise to typical or adaptive responses. To the degree that consensual emotion labels reflect normative evaluations, pattern recognition, or adherence to communicative norms, studies which employ the “validated” facial expressions are likely to observe far more unshared variance than shared variance between labels and responses—*not* because the scientific theory (whatever that theory is) of the *response* is inaccurate but rather because the facial stimuli were validated on the basis of labels that may fail to approximate the facial displays to which perceivers respond.

Ultimately, answers to the question raised in this section could engender conceptual clarity among scientists (*what* are we studying when we study emotion perception?). More importantly, evidence that labels for facial expressions are strongly driven by normative evaluation could lead to the labels people provide for facial expressions being weakly related with functional responses to facial expressions. Empirical answers to this question about the labeling process may be gathered via several different sorts of studies.

First, if participants are simply engaging in normative communication or pattern recognition when they label facial expressions, consensus in those labels should be roughly equivalent when those expressions appear on real human faces or on inanimate objects. Conversely, if participants do not simply engage normative labels or pattern recognition, and instead utilize some form of mental state inference, labeling consensus should be higher for real humans (who experience feelings) than for inanimate objects (that do not experience feelings). Studies which systematically vary the identity of the “emoter” between animate and inanimate and simply request emotion judgments (forced choice, Likert-ratings, open-ended) could test these hypotheses.

Second, it is possible to quantify the degree to which the labels that participants provide for facial expressions spontaneously reflect normative evaluations. Although many study designs may do so, a simple version would be a study with three conditions. Participants may report how *other people* would label each facial expression (normative condition), how they themselves would label each facial expression *disregarding what other people would label it* (personal condition), or be given standard instructions without any mention of “other people” (standard condition). To the degree that an emotion label for a facial expression is based on normative evaluations, (a) labels in the normative and personal conditions should be highly and positively correlated (have strong shared variance), (b) variance in the personal condition that is not shared with variance in the normative condition should fail to predict labels in the standard condition, and (c) variance in the normative condition not shared with variance in the personal condition *should* be predictive of labels in the standard condition. Such evidence would support the view that labeling methods for validation of facial expression are based on normative evaluations—not recognition of some internal emotional state—and may therefore be inappropriate stimuli for testing functional accounts of emotion perception.

## Concluding Remarks

Like with basic vision (e.g., Lupyan, 2015), research shows that emotion labels that perceivers provide for facial expressions have many downstream effects, shaping perceivers’ global impressions of, and behavior toward, a person (e.g., Gendron et al., 2012). Thus, such categorical labeling should be of interest to affective scientists. Moreover, understanding the visual processes that give rise to categorical labels for facial emotion can help scientists understand the antecedents to such consequential labeling. For some theories, verbal labeling is key, and the influence of verbal labels in felt and perceived emotion are clearly necessary. However, we argue that this approach should not be the sole method of pursuing the functional understanding of (a) how cognitive processing is influenced by others’ facial expressions, (b) how visual processing is shaped by exposure to facial expressions, (c) how social behavior is shaped by perceiving facial expressions; (d) the adaptive functions of perceiving facial expressions, and (e) ecological adaptations (perception-action) to facial expressions. In each of these cases, limiting “facial anger” (for example) to expressions that are normatively labeled as “anger” prevents adequate theory-testing. Process-oriented theories often regard *how* facial emotions condition perceivers’ visual, cognitive, affective processing. Tests of these theories typically use stimuli based only on *face validity*: if laypeople agree that an image should be labeled as “anger” then the image is said to depict an angry face.

The implications of this traditional approach are wide-ranging. First, this approach ensures that we scientists rely on laypeople to tell us (a) what the most meaningful emotions are and (b) what constitutes that emotion in a face. Yet, as implied by Adolphs and Andler (2018), this sort of approach prevents scientific discussion of what constitutes an emotion expression and more importantly, makes it more likely that effects may not be replicated in, say, 100 years: if facial emotions are defined by laypeople, new emotions can emerge and others may become less salient even if only due to new labels that emerge among a populace and labels that become less frequent in discourse. In this way, we scientists must then confront Gergen’s (1973) critique that social psychological experiments are largely historical analyses.

Some may *define* emotion perception in terms of multiple responses. For example, Adolphs and Andler (2018) argue that affective science (more generally) is unnecessarily shaped by the view that subjective “feelings” are equivalent to emotion. They argue instead that feelings are only one factor associated with a latent “emotion” variable, which is independently associated with certain perceptions, cognitions, physiologies, and action tendencies. Perhaps more relevant to the current discussion is the implication that psychologists have relied on lay terms to organize their concepts of emotion (“fear”, “anger”, etc.), rather than vice-versa. How many emotions have been examined by psychologists that do not match a lay term? But we can imagine an alternative approach that defines different emotions, by “clusters” of perceptions, cognitions, physiologies, and behaviors (and yes, “feelings”) that people typically (or individually) exhibit in response to different events.

## Footnotes

1 We endorse this definition but with an important caveat. Adolphs and colleagues argue against subjective experience as the primary criterion for emotion, but labeling may refer to subjective experiences as well as practical communication concerns, as in Gricean communication norms (see Schwarz, 1999). Accordingly, the label given to an emotion may not reflect subjective experience, and this is especially clear in the domain of emotion perception. For example, the facial expressions of artificially-generated avatars can be identified with emotion labels despite perceivers’ knowledge that these avatars have no internal experience of emotion—the labeled emotion presumably conforms to communicative norms for what to “call” the facial configuration. This caveat reinforces our arguments that verbally labeled emotions are suboptimal measures for many (functional) theories of emotion perception, and that validation procedures for facial expressions sets may simply draw from communication norms rather than the from mental state inferences.

## Additional Information

### Availability of Data and Material

No datasets were generated or analysed during the current study.

### Code Availability

No datasets were generated or analysed during the current study.
